# 338. ChatGPT Conveys Incorrect Information about the FDA’s Antibiotic Boxed Warnings

**DOI:** 10.1093/ofid/ofad500.409

**Published:** 2023-11-27

**Authors:** Rebecca Linfield, Julie Parsonnet

**Affiliations:** Stanford Health Care, Stanford, California; Stanford School of Medicine, Stanford, California

## Abstract

**Background:**

Chat Generative Pre-training Transformer (ChatGPT) is a popular artificial intelligence-based chatbot that rapidly provides responses to online inquiries. As a language-processing – rather than a scientific – tool, ChatGPT does not guarantee accuracy. The United States Food and Drug Administration (FDA) labels medications with significant risk of serious or life-threatening adverse effects with boxed warnings (BW). We sought to assess the accuracy of ChatGPT regarding FDA BW for antibiotics.

**Methods:**

FDA labels for commonly-used antibiotics were categorized through the DailyMed database of the National Library of Medicine (Table 1). ChatGPT-3.5 was then queried regarding FDA BWs using the following query: “Are there any boxed warnings on the FDA label for XX [medication] and if so, what are they?” Responses were sorted into 3 categories:

(1) “Matching”: ChatGPT accurately reported both existence and content of BW or its absence;
(2) “Inaccurate”: ChatGPT accurately reported a BW but erred on some aspects of the content; and
(3) “Incorrect”: ChatGPT misidentified existence of BW, or listed incorrect adverse effects (AE)

**Table 1:**
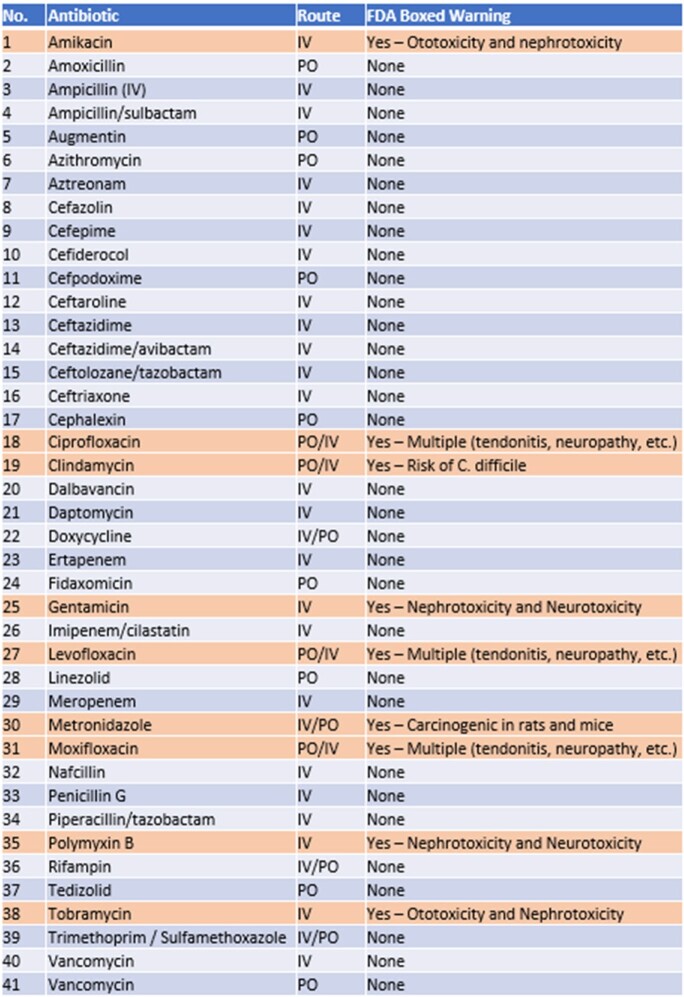
FDA Boxed Warnings for Commonly Used Antibiotics

**Results:**

Of the 41 antibiotics queried, 9 have FDA BWs and ChatGPT correctly identified the existence of all 9. However, ChatGPT correctly matched the AE of the FDA BW in only 3 instances (33%); in five instances (56%), ChatGPT listed inaccurate AEs (either under- or overinclusive) and in one (11%), ChatGPT listed an incorrect AE. For the 32 antibiotics without an FDA BW, ChatGPT reported that 23 (72%) had a BW (Figure 1). Figure 2 provides some examples of incorrect ChatGPT claims regarding AEs.

**Figure 1:**
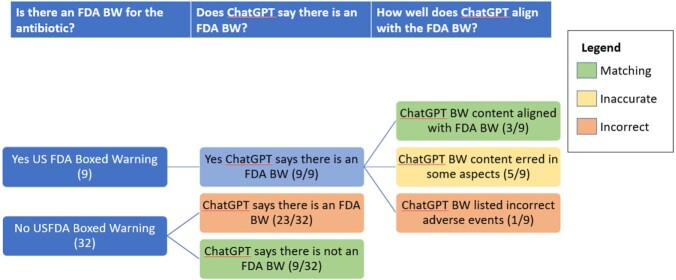
ChatGPT Responses about FDA Boxed Warnings

**Figure 2:**
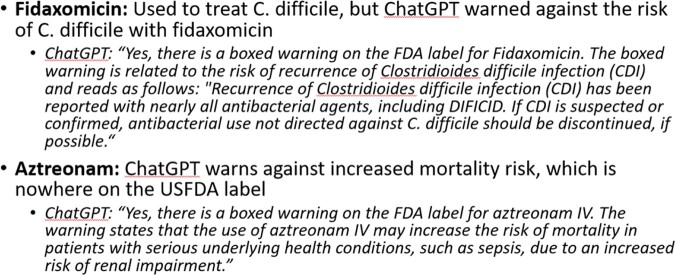
Examples of Incorrect ChatGPT claims

**Conclusion:**

ChatGPT reported inaccurate and incorrect information about FDA BW for antibiotics. Clinicians should be aware of this new source of information for patients.

**Disclosures:**

**All Authors**: No reported disclosures

